# How great is the medical burden of disease on the aged? Research based on “System of Health Account 2011”

**DOI:** 10.1186/s12955-017-0709-6

**Published:** 2017-07-03

**Authors:** Wenjuan Duan, Ang Zheng, Xin Mu, Mingyang Li, Chunli Liu, Wenzhong Huang, Xin Wang

**Affiliations:** 1 College of the Humanities and Social Sciences, China Medical University, No.77 Puhe Road, Shenyang North New Area, Shenyang, Liaoning Province 110122 People’s Republic of China; 2 Department of Breast Surgery, the First Affiliated Hospital of China Medical University, No.155 Nanjing Road, Heping Area, Shenyang, Liaoning Province 110001 People’s Republic of China; 3 The Hospital of Stomatology, China Medical University, No.117 North of Nanjing Road, Heping Area, Shenyang, Liaoning Province 110122 People’s Republic of China; 4 China Medical University, No.77 Puhe Road, Shenyang North New Area, Shenyang, Liaoning Province 110122 People’s Republic of China; 5 Library of China Medical University, No.77 Puhe Road, Shenyang North New Area, Shenyang, Liaoning Province 110122 People’s Republic of China; 6 College of Public Health, China Medical University, No.77 Puhe Road, Shenyang North New Area, Shenyang, Liaoning Province 110122 People’s Republic of China

**Keywords:** SHA 2011, Elderly, Curative care expenditure, Burden

## Abstract

**Background:**

The aging of population and the burden of disease among the aged have become one of the hot topics in the international health, and also brought tremendous pressure in the development of health service.

**Methods:**

A total of 1,377,681 patients aged 65 years and over were collected with multistage stratified cluster random sampling in 252 medical institutions in Liaoning China, and “System of Health Account 2011” was conducted to analyze the expenditure of disease for the elderly. Influencing factors were performed using multiple stepwise regression analysis.

**Results:**

The curative care expenditure for the aged was 233.18 billion RMB. Most of the expenditure for the old people was in hospital. Moreover, by the disease, the highest expenditure was incurred by non-communicable diseases. The financing scheme of the aged was concentrated on social health insurance and family health expenditure. Hospitalization expenditure was significantly associated with length of stay, operation, etc.

**Conclusions:**

This study intends to capture large data from various medical institutions with a new accounting system. The finding illustrates that the burden of old people is still heavy.

## Background

In recent years, the aging population and the burden of the aged have become some of the most popular topics in the world. About 23% of the burden of disease is attributed to people aged 65 and over [1]. In China, a developing country, the aging population problem is especially serious. By the end of 2014, 65 years and above population accounts for 10.1% of the total population [2]. On one hand, diseases in the elderly are mainly composed of non-communicable diseases (NCD), and the increasing of the prevalence of NCD in the people more than 65 years old is usually two to three times of the total population [3]. The substantial diseases in the old crowd lead to higher health care expenditure than other population. In 2012, among the elderly over the age of 65 in our country, health care expenditure is high, with 24.75% of the total health care expenditure [4]. The influence of aging on the growth of health care expenditure cannot be ignored [5]. In this study, we used a new system of health account, System of Health Account 2011 (SHA 2011), to account curative care expenditure (CCE) in the elderly. The CCE is different from health care expenditure, which refers to the expenditure of direct treatment that does not include the expenditure of prevention.

SHA 2011 could precisely count the burden of diseases in the elderly, and can better analysis population distribution on curative care expenditure, such as scale of curative care expenditure in the aged, fund raising, etc. In addition, SHA 2011 also could sufficiently analyze curative care expenditure in aspect of age-specific, sex-specific, and disease-specific on curative care expenditure [6, 7].

In China, total health expenditure has receded from 917.06 billion RMB in 2004 (1 USD ≈ 6.22 RMB, 2014)to 5748.58 billion RMB in 2014 (1 USD ≈ 8.27 RMB, 2014) [8]. The excessive growth of health expenditure turns out to be a hotspot in our society at present. How to solve the issue of “being unaffordable and difficult to see a doctor” has become the key point and difficulty in the course of our medical care reform [9]. Therefore, analyzing influencing factors of hospitalization curative care expenditure of 65 years and over patients is essential. Hospitalization curative care expenditure occupied the main part in medical institution, especially that in old folks [10]. But most researches focus on single disease or old people’s single disease, the study of all diseases of the elderly is rare.

## Methods

### Data source

Data was obtained from the *2015 Liaoning Health Statistical Yearbook, 2015 Liaoning Health Financial Yearbook, China National Health Accounts Report 2015, Liaoning Health Accounts Report 2015,* etc. Demographic data was obtained from *2015 Liaoning statistical yearbook*, medical institutions, and public health institutions.

### Study sample

The data used in our study was investigated with multistage stratified cluster random sampling. The first stage was to choose sample cities from Liaoning province based on taking full consideration into the perfection of health information management system and the level of economic development. Thus we selected Dalian, Liaoyang, Panjin and Tieling. The second stage was to select one county, one district, and then extracted three township hospitals and community health service organization in every country and district. Three village clinics and individual clinics were selected in rural town or community. The third stage was to choose medical institutions and public health institutions according to the type of institutions or administration structure. At last, seven provincial-level hospitals, 58 institutions in Dalian, 62 institutions in Liaoyang, 64 institutions in Panjin, 61 institutions in Tieling were selected. The basic information included gender, age, season, disease, expense, region, etc. The diagnosis of the diseases was coded according to the International Classification of Disease Tenth Revision (ICD-10). The sample of valid data was 1,377,681 after excluding the invalid or wrong message. The analysis of hospitalization care curative expenditure of 7185 cases for old patients whom we collected in Liaoning province was performed using multiple stepwise regression analysis, to find the main influencing factors of hospitalization curative care expenditure.

### Statistical method

#### Curative care expenditure accounted

Curative care expenditure (CCE) contained curative income^1^ and basic expenditure allowance,^2^ which refer to outpatient and inpatient. The formula was shown as follows;

1 $$ {S}_{CCE}=\sum_{k=1}\left({S}_{k INC}+{S}_{k ALL}\right) $$

In the above formula, *S*_*kINC*_ and *S*_*kALL*_ represent the curative income and basic expenditure allowance respectively in different medical institutions. The formula of curative income was as follows;

2 $$ {S}_{INC}={S}_{TINC}\left(1-\frac{a_p}{a}\right) $$

3 $$ {S_{INC}}^{\hbox{'}}=\sum_i^n\left({S}_{INC}\times \frac{a_i}{a-{a}_p}\right) $$

Above all of the formulas, curative income in per patient from the sample denoted as *a*_*i*_, total curative income denoted as *a*. Add all sample that contained preventive service on the basis of ICD-10, denoted as *a*_*P*_, then the sample’s curative income was *a*_*INC*_. Per patient sharing coefficient denoted by $\frac{a_i}{a-{a}_p}$ . Moreover, *S*_*TINC*_ represent the outpatient and inpatient’s income from *2015 Liaoning Health Statistical Yearbook* and *2015 Liaoning Health Financial Yearbook.* The purpose of formula (2) is to removed prevention expenditure in patient, and the formula of ${S}_{INC}\times \frac{a_i}{a-{a}_p}$ is to account the curative income in patient with the same age, gender, region, etc. The formula (3) means that could calculate curative income in various dimensions, such as age, disease, etc.

The formula of basic expenditure allowance was as follows;

4 $$ {S_{ALL}}^{\hbox{'}}=\sum_i^m\left[\left({S}_{TALL}-{S}_{PALL}\right)\times \frac{a_i}{a-{a}_p}\right] $$

Those formulas about basic expenditure allowance were similar to curative income, the *S*_*TINC*_, also collected from basic expenditure allowance from *2015 Liaoning Health Statistical Yearbook* and *2015 Liaoning Health Financial Yearbook.* The basic expenditure allowance in preventive service was collected from Yearbook too, denoted as *S*_*PALL*_. *S*_*TALL*_ − *S*_*PALL*_, means basic expenditure allowance. The formula (4), split the basic expenditure allowance to per patient, which means the patient with the same age, gender, region, etc. in curative income.

Curative income and basic expenditure allowance for old patient shared by above way. Mark off the different dimensions in age, gender, region, etc. for the elderly according the result., and the total curative care expenditure in different age, sex, disease, etc. all could account from above formulas (1).

### Financing scheme accounted

The calculation of the financing scheme in Liaoning must combine our actual condition. Therefore, the financing scheme was divided into the following types (Table 1). The calculation method was similar to that before.

**Table 1:** Financing scheme in China match the classification of financing scheme in SHA 2011

Classification of financing scheme in SHA 2011	Concrete financing scheme in China
HF.1 Public financing scheme	
HF.1.1 Government financing scheme	Medical assistance, emergency assistance, Medicaid, disability rehabilitation, other supplementary insurance
HF.1.2 Social health insurance	Urban employee ‘s basic medical insurance (UEBMI), Urban residents’ basic medical insurance (URBMI), New Rural Cooperative Medical System (NRCMS), Maternity insurance, Employment injury insurance, Endowment insurance, Unemployment insurance
HF.1.3 Mandatory medical savings account (CMSA)	No CMSA in China
HF.2 Voluntary financing scheme	
HF.2.1 Commercial health insurance	Commercial health insurance
HF.2.2 Non-profit organization financing	Charitable organization, Foundation, The Red Cross, etc.
HF.2.3 Enterprise and medical institution financing	
HF.2.3.1 Enterprise financing	Enterprise medical expenditure
HF.2.3.2 Medical institution financing	Income of medical institution
HF.3 Family health expenditure	Out-of-pocket payment
HF.4 Foreign financing scheme (non-resident unit)	Foreign medical institution’ health care financing for local residents

### Influencing factors of hospitalization curative care expenditure for old patients

The analyzed hospitalization curative care expenditure is used to find the main influencing factors of hospitalization curative care expenditure for the aged. The normal distribution test was conducted in the hospitalization curative care expenditure, because it was not in normal distribution. Then the abnormal distribution test was applied to analyze the data after logarithm transition. The data was normal distributed after logarithm transition. Influencing factors were performed using multiple stepwise regression analysis. Independent variable was age, gender, length of stay, hospital category, region, payment method, surgery. The enter criteria was 0.05, and exclusion criteria was 0.10. All statistical analyses were performed with SPSS software, version 22.0, and STATA software, version 12.0.

## Results

### Fundamental result in curative care expenditure for the aged

**Fig. 1:**
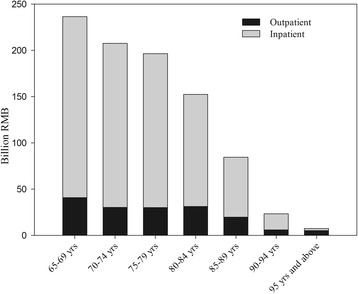
Distribution of outpatient and inpatient in different age group

The CCE in 2014 for 65 years and above was 233.18 billion RMB, accounting for 20.54% of total expenditure on health in Liaoning Province by source, and per capita was 4540.60 RMB, higher than per capita total expenditure on health (3028.54 RMB) [8]. The age group between 65 and 69 had the highest CCE, then declined in turn (Fig. 1).

### Allocation of medical institution for the aged in curative care expenditure

Most of the curative care expenditure for the old people was in the hospital (general hospital, traditional Chinese medicine hospital and special hospital), accounting for 92.96%, and the majority of the cost was in hospitalization expenses (83.77%). Basic medical institution was followed (6.66%). The expenditure occurring in the public health institution and ambulatory facility was 70,637.01 and 9285.63 thousand RMB respectively, rare in total expenditure. In general, the curative care expenditure in outpatient and inpatient accounted for 19.69% and 80.31% respectively. From the result, it is observed that the elderly spent a lot of CCE in the hospital rather than in other medical institutions. For example, basic medical and health institutions (township hospital, community health service center), ambulatory facility (village clinics, individual clinic) and public health institutions (maternity and child health organ) all had a small percentage of curative care expenditure. (Table 2, Fig. 1).

**Table 2:** Distribution of medical institution for the aged

	CCE (Billion RMB)	Outpatient (%)	Inpatient (%)
Hospital	216.78	16.23	83.77
General hospital	184.16	12.92	87.08
Traditional Chinese medicine hospital	30.94	35.05	64.95
Special hospital	1.68	32.00	68.00
Basic medical institution	15.53	63.67	36.33
Public health institution	0.77	96.64	3.36
Ambulatory facility	0.10	100.00	0.00
Total	233.18	19.69	80.31

### The expenditure of different disease

**Fig. 2:**
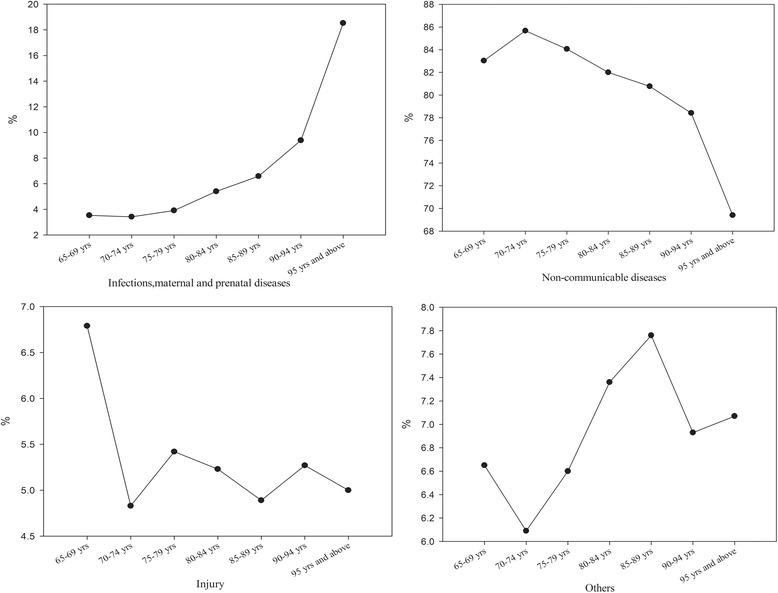
The CCE of diseases classified by GBD in different age

The Global Burden of Disease (GBD) made illness and injury divide into infections, maternal and prenatal diseases; non-communicable diseases; injury. Then on this basis, we added “others” according to the actual condition to reflect the illness and injury not classified (e.g. disease and syndrome of the traditional Chinese medicine diseases). In terms of GBD, the highest expenditure happened in non-communicable diseases. That showed the disease of the aged focused on non-communicable diseases and the age between 65 to 69 yrs. was responsible for the majority of expenditure. It may be the major part of disease for non-communicable diseases in the old population. But the distribution of disease had different performance in different age. The CCE in infection, maternal and prenatal diseasesrose with increasing age, but non-communicable diseases were opposite. Furthermore, the highest point of CCE in injury and others were between 65 and 69 yrs. and 85–89 yrs. respectively (Fig. 2).

**Fig. 3:**
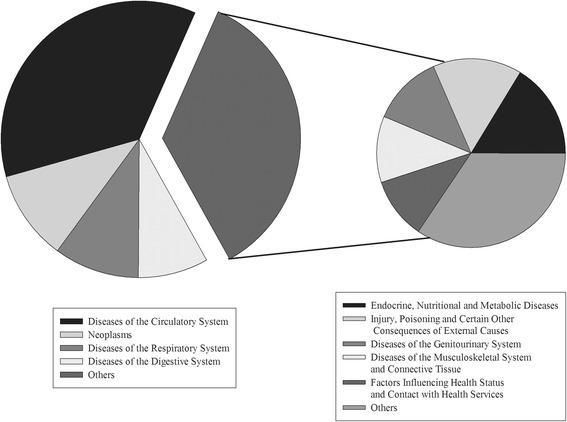
The CCE of diseases classified by ICD-10

Classified by the ICD-10, the top four of CCE in diseases were the circulatory system, neoplasms, diseases of the respiratory system, diseases of the digestive system, more than 60% in CCE, about 150.80 billion RMB. Besides, endocrine, nutritional and metabolic diseases were 13.43 billion RMB, also high in CCE (Fig. 3).

### Financing scheme in the aged

Financing in curative care expenditure was accounted as 75.79% for hospitalization service. Different financing scheme was different in outpatient and inpatient. Public health financing scheme and voluntary financing scheme were mainly in inpatient, but family health expenditure was opposite. That was owing to reimbursement mainly for inpatient of insurance, so most of the outpatient service was depended on family health expenditure.

**Fig. 4:**
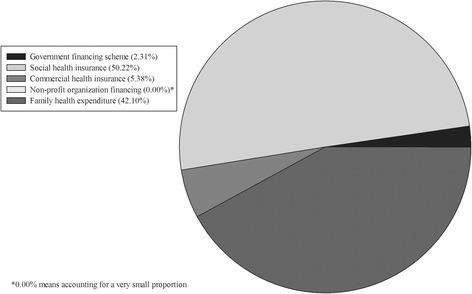
Financing scheme for the aged

The financing scheme of the aged was concentrated on social health insurance and family health expenditure (out-of-pocket payment (OOP)). Family health expenditure was accounted for 42.10% in total expenditure that reminds the burden of individual financing was heavy. Due to 65 years and older people retired in our country, they did not have enterprise financing. In addition, the financing was less in government financing scheme and commercial health insurance (Fig. 4, Table 3).

**Table 3:** financing scheme between inpatient and outpatient (%)

	Social health insurance	Government financing scheme	Commercial health insurance	Non-profit organization financing	Family health expenditure
Inpatient	73.85	49.77	100.00	100.00	23.70
Outpatient	26.15	50.23			76.30

### Influencing factors of hospitalization expenditure

The results of multiple stepwise regression analysis showed that,*F* = 1987.499*P* = 0.000*,* the regression equation was established. From the standard partial regression coefficient, the top three factors influencing hospitalization expenditure were the length of stay, operation and region (development of the region) (*P* < 0.005), while gender was excluded. And the *R* ^2^ = 0.862 after corrective. That means all the independent variable, which incorporated into the equation, could account for 61.5% to the dependent variable degree of variation (Table 4).

**Table 4:** Influencing factors analyzed by multiple stepwise regression

Model	Unstandardized Coefficients		Standardized Coefficients	t	Sig.
	B	Std.Error	Beta		
Length of stay	.061	.001	.570	83.629	.000
Operation	.618	.010	.430	62.810	.000
Region	−.686	.014	−.323	−48.283	.000
Age	.006	.001	.040	5.971	.000
Insurance	−.067	.014	−.032	−4.822	.000
Season	.013	.004	.022	3.407	.001

## Discussions

We comprehensively analyzed curative care expenditure of 65 years and older based on SHA 2011, including distribution in age, disease, medical institution or reflected financing, origin and destination. Moreover, influencing factors were performed by multiple stepwise regression analysis.

### The distribution of medical resources in medical institutions in the aged

From the result of SHA 2011, it is observed that the elderly spent a lot of CCE in the hospital rather than other medical institutions. For example, basic medical and health institutions (township hospital, community health service center) as well as ambulatory facility (village clinics, individual clinic), public health institutions (maternity and child health organ) all had a small percentage of curative care expenditure (Table 2). Furthermore, the other problems are the distribution of health resources is unreasonable, and health resources are deficient in the rural area [11]. Medical resources prefer to the place where hospitals have huge purchasing power and high-level surgical treatment or high drug profit. Basic medical and health institutions, however, lack of medical workers, financial and other resources, which make the senile patients, more willing to go to a hospital. Peter’s analysis of Europe’s decades of medical reform thought to think promoting priority resources allocation ability should rely on evidence-based medicine reviews (EBMR), goalkeeper physicians (GP), performance reporting system (PRS) etc [12].

The number of health institutions and health technicians in Liaoning province are in medium level in the China. However, in 2014, there were very few government health investments in Liaoning province (the government health expenditure made up 21.7% of the total health expenses), which the government health expenditure were 29.96% in the total health expenses in China [8]. Changing the distribution of medical resources and solving problem of payment mechanism are necessary. If the new round of health care reform wants to achieve universal coverage of basic medical services in 2020, it requires us to consider the accessibility and affordability of medical service, and research on priority resources allocation is contributing to the sustainable development of our country’s medical system [13].

We found that old patient’s hospitalization expenditure took the largest proportion in curative care expenditure. It is because the major part of disease for non-communicable diseases in the old population (Fig. 2). This kind of disease treatment is lengthy and difficult,which lead to high hospitalization expenditure. Moreover, some study shows that inpatient expenditure will be higher than outpatient expenditure with the same disease in the elderly patients [14]. It is also related to the proportion of reimbursement in new medical reform. In China, taking Liaoning urban worker’s basic medical insurance for an example, the outpatient reimbursement ratio is lower than the inpatient ration and some special drug or examination fee are not reimbursed in outpatient but only in inpatient, which makes the aged patients try to transfer from outpatient to inpatient. Such phenomenon not only increased the hospitalization expense but also wasted the national health resources seriously [15].

### The burden of disease in the aged

The results of the study showed that the per capita curative care expenditure of the aged was much higher than that of other groups, which means the heavy burden is on old people. Not merely in China, but also in other developing and even developed countries, there is also heavy burden on the elderly.

The main cause of severe burden of disease on the old people was diseases of cardiovascular system (30.3%), malignant neoplasm (15.1%) etc. [1] that is in accordance with our research. We can effectively control the cost of the disease if triple prevention is applied for elderly people. For the aged who has serious illness, the government, can provide a suitable prognostic measure to prevent further deterioration and improve the overall outcome for the individual. Priority is given to shelter the high-risk elderly, which could cut length of stay and hospitalization expenditure [16]. Furthermore, Sonya Haw [17] suggests that prevention and health care, especially in the early prevention, not only improve life quality but reduce greatly the burden on society, families and individuals. The demand of old people for prevention and health care is higher than that of young people, but the level of health care is not perfect in China where the health care institutions and workers are in deficiency [18]. In conclusion, strengthening the construction of preventive health care team and institution is urgent, as well as giving appropriate compensation in purchase preventive health care serves.

Firstly, many studies of diseases of the old have illuminated the diseases of the circulatory system resulted in unpredictable costs. The WHO believes that the cardiovascular disease (CDV), the main circulation system disease, is one of the most popular and dangerous disease in the world. In China’s most prosperous cities, the health care expenditure per capita which is more than 25,000 RMB, and the medical cost for old population are much higher [19]. As the risk factors of circulation disease continue to grow rapidly, such as obesity, high-fat-diet, high salt diet, etc., it will aggravate the current epidemics of diseases of the circulatory system and lead to high expense.

Secondly, we found that the CCE was also high in neoplasm for the aged which ranked second and was behind the diseases of the circulatory system. The standard incidence of malignant neoplasm is from 0.527% in 1990 to 0.750% in 2010 [20]. But the accurate CCE is still unclear. In this research, the CCE of neoplasm is 13.43 billion RMB, much more than other populations. Elder cancer researchers focused on the incidence and the nursing that also shows the incidence of senile neoplasm higher than young people [21]. However, there is dissimilarity in the country using different nurse methods. In developed countries, good care is the key to decrease neoplasm curative care expenditure. In China, the important reason of the costs of neoplasm at a high level all the time is improper nursing that would aggravate disease. The aged who has a malignant neoplasm, the rate of hospital infection among chemotherapy and radiotherapy can reach 44.36% [22]. Those expenses caused by infection are also counted as a part of neoplasm’s curative care expenditure. Neoplasm treatment cycle is long and is also the important reason for the high curative care expenditure in neoplasm. In short, strengthening nursing care for senile neoplasm during hospitalization and treatment has become the crucial measure to reduce the expense.

Thirdly, the burden of respiratory system disease ranked third following circulation system and neoplasm diseases in Liaoning. That is because Liaoning, the heavy industry base in north China, has built a lot of polluting factories, which produce dirty air seriously. Furthermore, winter-time in Liaoning is long and very cold that has a six-month heating period each year. Some study showed that the concentration of PM 2.5 increased year by year and was significantly higher in the heating period (from November of the year to April of the next year) compared to that of other months. With the increased proportions of 11.6%,18.9%,and 35.8% for the year of 2009 to 20 l1 and the PM 2.5 concentration in the heating period of the 3 year was 44,35,and 60 *μg*/*m* ^3^, respectively [23]. And the 65 years and older people are extremely sensitive to air pollution [24]. In the developed region, patients with respiratory diseases, the per capita expense could be up to 2878 RMB. In short, controlling the expenditure in respiratory system disease in the heating period is the key to lower the cost for the aged.

The CCE of diseases of the digestive system and endocrine, nutritional and metabolic diseases were heavy in elderly people too. That is related to traditional eating habits of the aged. Plenty of them prefer pickled foods and high-fat foods. Those bad eating habits lead to the morbidity of digestive disease and nutritional and metabolic diseases rising continuously, which brings high medical expense. Some study showed the direct cost of diabetic patients was four times than that of who did not have diabetes. Those expenses mainly were used to take care of the old and for hospital care [25]. Not only expenditure of diabetes, as well as morbidity of digestive disease and nutritional and metabolic diseases all were heavy burdens.

These diseases have characteristics in a long cycle of treatment as well as in special treatment groups. We can establish comprehensive single payment disease system that could cover the high curative care expenditure of diseases of old people who have registered in the basic medical insurance system. The basic medical insurance system in Liaoning province has included single payment disease system this year. In addition, with the development of critical illness insurance in recent years, it is observed that many poor families can avoid catastrophic health expenditure because of critical illness insurance. In China, some areas have performed critical illness insurance system, but the same with single payment disease system, have not been universal.

### The distribution of financing scheme in the aged

In this research, we analyzed simultaneously the distribution of the financing scheme in the elderly. We could best understand the distribution of financing in the aged.

The health financing calculation based on SHA 2011 has included all of the health subsidy and types of health insurance. The 65 yrs. and older have retired, so we did not include them. The social financing accounted for major curative care expenditure in all financing schemes. Family financing scheme, also called OOP, followed the social financing scheme. Moreover, there was a high proportion in OOP of the aged. The measurement results of OOP in Liaoning province by the method of health financing resource showed that OOP accounted for the total health expenditure was 36.04% [26]. But in our research, OOP in the aged was 42.10%, which means expenditure burden was severe in the aged. From the distribution of outpatient and inpatient in health financing, we knew the elderly’s financing focused on inpatient. That was because long hospital stay and higher hospitalization expenditure led to high compensation cost.

Although health insurance in China can cover most of the old people, the outcome is not positive. Equity in health financing is set as a main objective by the global health policy. Many countries are trying to control the ever-increasing of public financing that make the OOP unceasingly increase. Rural and urban area’s patients in India have poverty health care financing are 40% and 60% respectively [27]. The Portuguese scholars believed that the huge medical expenses were in urgent need to cut down that used to risk resistance in elderly people cannot afford [28]. Our preliminary research showed that the government had increased the health investment from new medical reform, but individual was still the main undertaker of health expenditure [13]. Hence the health financing structure should be adjusted immediately, dominated by public financing and concentrating on equity of China health financing among developed provinces and less developed provinces.

Almost all of the aged who are unable to work and lack of guaranteed by society, and they must be supported by their children, which makes the young family undergoes considerable stress. So, endowment insurance needs to be expanded. And designing specific type of insurance for the aged is to increase reimbursement when they experience disease. In our research, the proportion of commercial insurance financing is very low. The commercial insurance market of our country develops relatively late and far less than developed countries. What’s more, the reimbursement of commercial insurance is aimed at inpatient, rarely in outpatient. The government should introduce appropriate policies to encourage the development of commercial insurance particular for major medical expenditure, and not only in hospitalization, but in outpatient who needs medical insurance as well.

### Control of the hospitalization curative care expenditure in the aged

The result of stepwise regression analysis showed that, the topic factors influencing hospitalization curative care expenditure included length of stay, operation, region, age, insurance and seasons. All factors were within the controllable range except age. The biggest impact factor was hospitalization days that maybe related to improvement of medical condition and enhance of elderly people’s health awareness, which led to admission number increased rapidly. The longer hospitalization days, the higher curative care expenditure would be. As this studied in many researchers, it was also accordant with our research [29–31]. The operation is another key factor influencing hospitalization curative care expenditure [32]. The cost of materials is the main composition of operation fee. The non-communicable diseases (NCD) need expensive imported surgical materials, and account for a very large proportion in disease among the aged. Thus surgeons tend to use imported surgical materials so that the risk of operation could be reduced, which ultimately makes the high medical expenses [33]. In our study, we found that the influence of insurance for hospitalization curative care expenditure was not very big. The high proportion of elderly OOP caused a little effect of insurance in the process of seeking medical care. So, there is an urgent need for government to take competent measures to reduce the proportion of OOP in the aged. In addition, economic development level in China caused different hospitalization curative care expenditure level of individual old residents. However, this is the same as what happened in other countries [34].

The research indicated that in the hospitalization curative care expenditure, the drugs’ income accounted for most of the total expenditure. As far as character in China medical system, expenses for medical have become a main factor for hospitalization expenditure. Medical worker’s value is ignored for a long time that brings about them taking rebate from prescription to make many illegal interests, which promote problem of “see a doctor expensively”. That showed the drugs addition policy in new medical reform need further improvement. Therefore, it needed to be transferred and controlled the drug expenditure, and the composition of hospitalization expenditure should be adjusted. The medical worker’s page is in urgent need of increasing as well [35].

Shortening days of hospitalization is part of the most effective ways to control the medical expenses. To check the irrational increases of medical expenses, we must reinforce the administration of rational prescription and examination and farther consummate the reform of the medical care system. We could ultimately reduce the hospital costs of patients as well as the economic burden of patients and society, by strengthening the hospital management, shortening hospital stay, and rationally regulating drug use.

## Conclusions

The curative care expenditure in 65 years and above is accounting for 20.54% of total population in Liaoning. And the per capita expenditure was also higher than per capita total expenditure on health. The highest expenditure was incurred in non-communicable diseases classified by GBD. Family health expenditure is also heavy in total expenditure. Besides, the top three factors influencing hospitalization expenditure were the length of stay, operation and region.

## Abbreviations

CCE: Curative care expenditure
GBD: Global Burden of Disease
GDP: Gross Domestic Product
OOP: Out-of-pocket
SHA 2011: System of Health Account 2011

## Footnotes

1 Directly expenditure for the patients to see a doctor, including treatment fees, drug fees, examination fees, etc.

2 Government subsidy to the hospital when the patient to see a doctor.
